# Mechanisms of exercise for diabetic neuropathic pain

**DOI:** 10.3389/fnagi.2022.975453

**Published:** 2022-10-12

**Authors:** Jing Luo, Hui-Qi Zhu, Bo Gou, Yi-Li Zheng

**Affiliations:** ^1^Department of Sport Rehabilitation, Xian Physical Education University, Xian, China; ^2^Department of Sport Rehabilitation, Shanghai University of Sport, Shanghai, China; ^3^College of Kinesiology, Shenyang Sport University, Shenyang, China

**Keywords:** exercise, diabetic neuropathic pain, mechanism, therapy, review

## Abstract

Diabetic neuropathic pain (DNP) is a common disease that affects the daily lives of diabetic patients, and its incidence rate is very high worldwide. At present, drug and exercise therapies are common treatments for DNP. Drug therapy has various side effects. In recent years, exercise therapy has received frequent research and increasing attention by many researchers. Currently, the treatment of DNP is generally symptomatic. We can better select the appropriate exercise prescription for DNP only by clarifying the exercise mechanism for its therapy. The unique pathological mechanism of DNP is still unclear and may be related to the pathological mechanism of diabetic neuropathy. In this study, the mechanisms of exercise therapy for DNP were reviewed to understand better the role of exercise therapy in treating DNP.

## Introduction

The incidence rate of diabetes worldwide is very high and the number of individuals with diabetes will increase to 642 million by 2040 (Ng et al., 2021). Diabetes affects 8.3% of Americans, and the annual treatment cost of diabetes is US$ 174 billion (Feldman et al., 2019). Diabetic neuropathy is the most common chronic complication in patients with type 1 or 2 diabetes (Javed et al., 2015; Melese et al., 2020) and accounts for 54–59% and 45% of type 1 and type 2 diabetic patients (Zilliox and Russell, 2011), respectively. Pain, abnormal sensation, and decreased stability are the typical clinical symptoms of diabetic neuropathy (Goldberg et al., 2008; Marcovecchio et al., 2010).

The diabetic neuropathic pain (DNP) associated with diabetes is gradually increasing with the prevalence of diabetes (Tesfaye et al., 2010). It is reported that 25% of patients with type 2 diabetic neuropathy will experience pain (Callaghan et al., 2015). In general, symptoms related to DNP are burning, lacerating, tingling and shooting pain, loss of protective sensation, and hyperalgesia (Kirk et al., 2019). DNP hurts patients’ quality of life compared with painless diabetic neuropathy (Van Acker et al., 2009).

Currently, there are no treatments available to completely cure DNP; however, drugs and exercise are its common symptomatic treatments (Pop-Busui et al., 2017). There are three main categories of drugs used to treat DNP: antiepileptics, antidepressants, and non-specific analgesics (Feldman et al., 2019). However, the severe side effects and high cost of drug therapy have greatly limited the treatment of DNP (Tölle et al., 2006). Exercise for pain has received increasing attention in recent years, i.e., exercise for low back pain (Peng et al., 2022). Research on mechanisms related to exercise for pain has also been favored by researchers, i.e., effects of exercise-induced hypoalgesia (Wu et al., 2022). Meanwhile, the effectiveness and safety of exercise have been proven for people with diabetes (Lemaster et al., 2008; Chiang et al., 2016). According to the American Diabetes Association proposal, at least 150 sessions of moderate to vigorous aerobic exercise for adults with diabetes (60 min for children and adolescents) and 2–3 sessions per week of resistance training are need (American Diabetes Association, 2018). More randomized controlled trials are needed to further explore the mechanisms by which exercise reduces DNP in order to select the appropriate exercise prescription for treatment. This review describes the pathological mechanisms associated with exercise for DNP based on recent studies on exercise for DNP.

## Mechanisms of exercise in improving diabetic neuropathic pain

**FIGURE 1:**
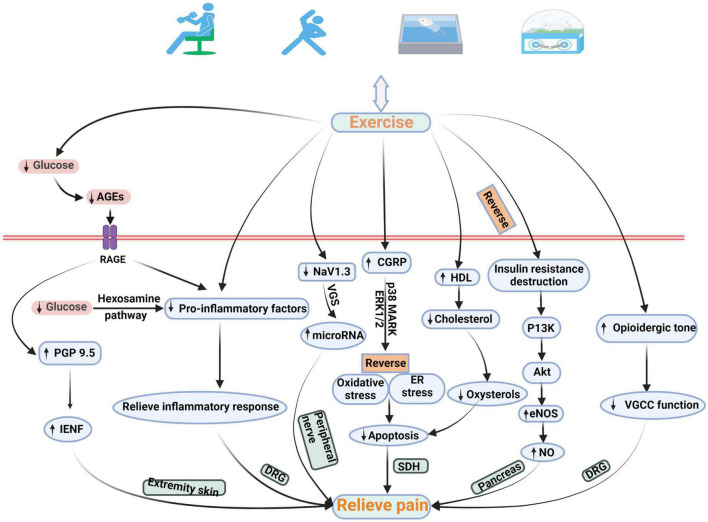
Schematic diagram of the possible pathological mechanism of exercise in the treatment of DNP. Exercise relieves DNP by changing the quantity of IENFs, expression of pro-inflammatory factors, NaV1.3, CGRP, HDL, and eNOS, and VGCC function. AGEs, advanced glycation end products; IENF, intra-epidermal nerve fiber; CGRP, calcitonin gene-related peptide; ASK1, apoptosis signal-regulating kinase 1; p38 MARK, p38 mitogen-activated protein kinase; ERK 1/2, extracellular signal-regulated kinase ½; ER, endoplasmic reticulum; PI3K, phosphatidylinositol-3-kinase; eNOS, endothelial nitric oxide synthase; VGCC, voltage-gated calcium channel.

Exercise is an integral part of DNP management plan, and it improves blood glucose control, reduces cardiovascular risk factors, reduces obesity, and promotes good health (American Diabetes Association, 2018). Doing exercise for treatment of DNP is increasingly favored by public-health worker. This paper summarizes studies related to the pathological mechanisms of exercise therapy for DNP (Table 1). This is different between exercise can reduce pain and exercise can reduce neuropathic pain in diabetes. Given that the unique mechanism of pain production is unclear, the pathological mechanism of diabetic neuropathy may be related to that of DNP (Calcutt, 2020). Therefore, it was concluded from the pathological mechanism of diabetic neuropathy that exercise may relieve pain related to DNP by affecting blood glucose and intra-epidermal nerve fibers (IENFs), inflammatory response, miRNA, apoptosis, deficit in oxide synthase (NO) synthesis, and dysregulation of voltage-gated calcium channels (VGCCs) (Figure 1).

**TABLE 1:** The possible pathological mechanism of exercise in the treatment of DNP.

Reference	Model	Exercises types	Related gene/cytokines/protein/ion	Involved in pathways/channel	Functions	Change
Nadi et al. (2019)	DNP Patients model	Resistance and peripheral neuropathy exercise	TNF-α, IL-10, CRP, HbA1c		Inflammation, Glucose	↓
Heidari et al. (2021)	DNP Patients model	Integrated exercise	HbA1c		Glucose	↓
Yoo et al. (2015)	DNP Patients model	Aerobic exercise	HbA1c		Glucose	↓
Ma et al. (2019)	DNP rat model	Treadmill exercise	IL-1β, IL-6, TNF-α		Inflammation	↓
Nascimento et al. (2012)	DNP rat model	Treadmill exercise	Calcitonin gene-related peptide		Apoptosis	↑
Shankarappa et al. (2011)	DNP rat model	Treadmill exercise	Ca2^+^	LVA T-/HVA N-type	Dysregulation of VGCC	↑
Aghdam et al. (2018)	DNP rat model	Swimming exercise	NaV1.3	VGS	miR-96	↓
Ma et al. (2020)	DNP rat model	Treadmill exercise	p-S6K1, p-4E-BP1, IL-6	IL-6-mTOR	Inflammation	↓
Jensen et al. (2019)	DNP patient model	Aerobic and resistance exercise	HbA1c		Glucose	↓
Chen et al. (2013)	DNP rat model	Swimming exercise	TNF-α, IL-6, Hsp72		Inflammation	↓
Olver et al. (2014)	DNP rat model	Treadmill exercise	eNOS	PI3k-Akt	NO synthesis	↑
Yoon et al. (2015)	DNP rat model	Treadmill exercise	TNF-α, IL-1β, Enkephalin, Hsp70	TRPM8, TRPV1	Inflammation	↑
Kluding et al. (2012)	DNP patient model	Aerobic and resistance exercise	HbA1c, PGP9.5		IENF	↑
Groover et al. (2013)	DNP mice model	Treadmill exercise	PGP9.5, TrkA, GDNF, NGF, BDNF		IENF	↑
Lohmann et al. (2010)	DNP patient model	Physical activities	HDL, HbA1c		Apoptosis, Glucose	↓
Chen et al. (2015)	DNP rat model	Treadmill exercise	IL-10, IL-6, TNF-α		Inflammation	↓

### The role of exercise in glucose control and intra-epidermal nerve fibers

It is known that hyperglycemia is an essential factor that causes diabetes, and that extended hyperglycemia can cause cellular damage (Callaghan et al., 2012). The pathogenesis of DNP remains to be unknown, and hyperglycemia leading to production of advanced glycation end products (AGEs) may be one of the mechanisms for the emergence of pain (Sugimoto et al., 2008). In this signal pathway, glycation of extracellular matrix protein laminin leads to impaired regenerative activity in diabetic neuropathy (Sugimoto et al., 2008). AGEs connect reactive carbohydrate groups to proteins, lipids, or nucleic acids to affect cellular functions (Duran-Jimenez et al., 2009), and AGEs bind to their receptor, initiating NADPH oxidases and generating oxidative stress further damaging neurons, vascular endothelial cells, and thus, leading to reduced IENFs (Vincent et al., 2007; Boric et al., 2013; Do et al., 2020). To quantify intraepidermal nerve fiber density, biopsy of the hairy skin in the lower extremities is a more commonly accepted method (Peltier et al., 2014; Lauria et al., 2010).

A test was conducted to clarify the effects of exercise on controlling the glucose level of diabetic patients. Jensen et al. (2019) conducted 12 weeks of exercise on 50 diabetic patients with musculoskeletal pain, and the results indicated that exercise reduced pain, and that HbA1c (mmol/mol) was decreased from 60 ± 15 to 54 ± 11. These results suggest that exercise effectively improves glucose control in diabetic patients’ neuropathy. Therefore, it is theoretically feasible that exercise can reduce IENF damage by improving blood glucose. Ten weeks of aerobic and resistance exercise interventions on 17 patients with diabetic peripheral neuropathy was conducted by Kluding et al. (2012) After the program, the patients were processed for immunohistochemistry of the right leg proximally at the lateral thigh and distal part of the lateral ankle using a rabbit anti-PGP9.5 primary antibody, and the IENF density was quantified. It was found that exercise significantly reduced pain, lowered HbA1c, and increased PGP 9.5 in the epidermis of the patients, and that the number of branches per fiber in the proximal biopsy site had improved. However, there was no significant increase found in the distal IENF measures or proximal IENF density, and this may be due to some subjects being pain-free before the exercise intervention, limitations of current guidelines on IENF quantification, and testing of IENF density at different time points before and after the exercise intervention (Kluding et al., 2012). Therefore, the specific role of IENFs in DNP needs further study or guidelines to quantify the IENF density to be more detailed and understood. Contradictory findings regarding IENFs in patients with DNP have been reported (Smith et al., 2001; Polydefkis et al., 2003). Another study explained the pathological mechanism of exercise intervention in DNP in terms of neurotrophin and absence of significant change in IENFs in DNP. Groover et al. (2013) developed a DNP mouse model by giving the male C57Bl/6 mice a high-fat diet and then quantifying the IENFs using a rabbit anti-PGP9.5, and the growth factor using an ELISA kit. The quantification of IENF density showed that the high-fat diet and exercise changed the number of PGP9.5-immunopositive fibers. However, the percentage of TrkA/PGP9.5 fibers was 30% lower in the exercise group than in the sedentary control group. The related growth factor and nerve growth factor (NGF) were significantly decreased. Meanwhile, brain-derived neurotrophic factor (BNDF) and glial cell line-derived neurotrophic factor (GDNF) were significantly increased (Groover et al., 2013). The increase in TrkA axons in the DNP mouse model can mediate the increase in NGF (Kashiba et al., 1996), and NGF has an important role in pain management (Pezet and McMahon, 2006). Therefore, this is one of the pathological mechanisms of DNP, and exercise also reduces pain related to DNP by decreasing NGF and TrkA axons. In addition, TrkA-expressing fibers are peptidergic fibers that express nociceptive neuropeptides (Kashiba et al., 1996), and non-peptidergic fibers are sensitive to GDNF (Groover et al., 2013). Therefore, the absence of change in IENFs after exercise intervention in DNP may be due to a decrease in peptidergic fibers and increase in non-peptidergic fibers. However, to determine specific non-peptidergic fibers changes, they should be quantified by markers.

### The role of exercise in inflammatory response

Under normal physiological conditions, inflammatory response is the immune system’s response when a harmful stimulus causes damage to the body (Weiss, 2008). In recent years, evidence has been emerging from related research that inflammatory response has a significant role in the pathogenesis of DNP (Pop-Busui et al., 2016). For example, increased pro-inflammatory factors tumor necrosis factor-α (TNF-α), interleukin-10 (IL-10), and C-reactive proteins (CRPs) lead to an inflammatory response associated with neuropathy in diabetes (Shoelson et al., 2006; Pop-Busui et al., 2016). Galloway and Chattopadhyay (2013) constructed the DNP rat model and observed the expression of pro-inflammatory factors and found that compared to rats with no pain, overexpression of a large number of pro-inflammatory factors in NDP rats such as TNF-α, IL-1, 6, 13, and 17, chemokines such as MIP1 and 3, Regulated on Activation, Normal T Cell Expressed and Secreted (RANTES), Fractalkine, and cell adhesion molecule sICAM in dorsal root ganglion (DRG) that are associated with pain phenotype remarkably increased. These results suggested that the pro-inflammatory factors in DRG play a non-negligible role in DNP (Galloway and Chattopadhyay, 2013).

Exercise inhibits inflammatory response by reducing pro-inflammatory factors, thus, reducing pain in DNP, which is the common activity mechanism in treating DNP. Ma et al. (2019) established a DNP rat model by intraperitoneal injection of streptozotocin (STZ) and made DNP rats perform exercise on a treadmill. Paw withdrawal thresholds (PWTs) were significantly increased in the STZ rats by the exercise compared to the control group, and relief of pain was accompanied by decreases in levels of IL-1β, IL-6, and TNF-a. This suggests that exercise modulates pro-inflammatory factor signaling pathways, hence reducing the inflammatory response and further inhibiting DNP. Furthermore, blocking individual pro-inflammatory factor receptors elevated PWTs. This result illustrated the side effects of inflammatory response on regulation of DNP (Ma et al., 2019). Similarly, Yoon et al. (2015) exerted moderate-intensity exercise on DNP rats and they used the level of TNF-α and IL-1β in the DRG as the observations. The results showed that the exercise inhibited hyperalgesia and recess the development of cold allodynia through the TRPM8 and TRPV1 ion channels. Meanwhile, immunohistochemical studies on DRG also showed a significant decrease in TNF-α and IL-1β in the diabetic exercise group. It is an important mechanism in changing the pain threshold in DNP test animals (Yoon et al., 2015). Interestingly, an exercise intervention study on hyperalgesia and allodynia in DNP rats showed that exercise reduced diabetic-associated thermal hyperalgesia and increased heat shock protein 72 (Hsp72) (Chen et al., 2013). Hsp72 has a neuroprotective role in neuropathic pain in rats after peripheral nerve injury (Chen et al., 2012). However, unlike the above study, the expressions of TNF-α and IL-6 have no difference between the exercise and sedentary groups after the exercise intervention, which may be due to the acute pro-inflammatory effect of the short-term exercise (only 2 weeks of exercise intervention). This is because the acute pro-inflammatory effect after each exercise session causes a transient increase in pro-inflammatory factors such as TNF-α, IL-1β, and IL-6 (Nemet et al., 2002; Galassetti et al., 2006; Chen et al., 2013). However, further investigation is needed to know the specific mechanistic relationships among exercise duration, pain, and pro-inflammatory factors. In conclusion, exercise can modulate DNP inflammatory factors and suppress inflammatory responses, consequently reducing pain in DNP.

### The role of exercise in micro-ribonucleic acids

Micro-ribonucleic acids (microRNAs) are short categories of non-coding RNAs and they regulate the expression of their target genes by repressing protein translation (Bartel, 2004; Winter et al., 2009). Previous studies have shown that abnormal expression of microRNAs in white blood cells, the sural nerve, and skin is associated with painful peripheral neuropathies (Leinders et al., 2017). Chen et al. (2014) showed that enhanced NaV1.3 expression exacerbated neuropathic pain, and that among microRNAs, miR-96 alleviated neuropathic pain by decreasing the expression of NaV1.3.

Aghdam et al. (2018) established a DNP rat model by intraperitoneal injection of STZ and with a high-fat diet for 1 month to elucidate the effect of exercise on miR-96 and NaV1.3. Rats were subjected to 10 weeks of swimming exercise, after which real-time quantitative PCR was conducted to measure the expression of miR-96 and NaV1.3. The results showed that the swimming exercise group’s thermal pain threshold was remarkably prolonged compared with that of the control group. The swimming exercise reversed the enhanced expression of NaV1.3 caused by diabetes, whereas increasing expression levels and the swimming exercise decreased NaV1.3 expression in the healthy rats. These results indicated that miR-96/NaV1.3 mRNA is a possible mechanism for a protective effect of swimming exercise on diabetic thermal hyperalgesia. In healthy rats, exercise can regulate pain related to DNP through gene regulation pathways (Aghdam et al., 2018).

### The role of exercise in apoptosis

Apoptosis is an automatic, evolutionarily conserved, and genetically determined form of programmed cell death that is necessary for maintaining homeostasis and growth in animal tissues (Cheng and Ferrell, 2018). Pancreatic β cells, which are responsible for insulin production, are associated with ER stress-induced apoptosis caused by oxidative stress in diabetic neuropathy. The main action pathway is the apoptosis signal-regulating kinase 1 (ASK1)/p38 mitogen-activated protein kinase (MAPK) signaling pathway (Callaghan et al., 2012; Yamaguchi et al., 2013). Inhibition of the ASK1 signaling pathway reduces the chances of apoptosis pancreatic β cell death (Pepin et al., 2014). In general, the role of calcitonin gene-related peptide (CGRP) in diabetic patients is to control blood glucose, thus reducing the release of insulin (Russell et al., 2014), and insulin increase in diabetic rats is accompanied by loss of CGRP-containing fibers (Hashikawa-Hobara et al., 2012). In an earlier study, CGRP was localized in pancreatic β cells (Westermark et al., 1987), and CGRP prevented apoptosis of vascular smooth muscle cells caused by oxidative stress by activating the extracellular signal-regulated kinase 1/2 (ERK1/2) and p38 MAPK signaling pathways (Schaeffer et al., 2003). Therefore, the mechanism of apoptosis associated with CGRP is involved in the pathological mechanism of DNP. An STZ injection-induced diabetic rat model was established by Nascimento et al. (2012) to research on the role of exercise in DNP and CGRP levels in DRG. A tail-flick apparatus was used to assess nociceptive injury changes. Meanwhile, the immunohistochemical procedure and optical densitometry were performed to measure the level and intensity of CGRP. The results showed that exercise reduced the hyperalgesia and reversed the diabetes-induced decrease in CGRP in the DRG levels of diabetic rats (Nascimento et al., 2012). Therefore, the exercise mechanism for nociceptive hyperalgesia may be increase in CGRP, which reverses the pancreatic β-cell death induced by oxidative and ER stresses through ASK1, p38 MARK, and ERK1/2. That is to control the release of insulin, and then control blood glucose, and finally reduce pain related to DNP. In addition, compared with before the exercise intervention, another study has found an 8.6% increase in HDL cholesterol after the exercise intervention (Lohmann et al., 2010). HDL decrease and LDL increase are inducing factors of oxidative stress, and cholesterol can lead to apoptosis when oxidized to oxysterols (refer to Callaghan et al., 2012 for details). Therefore, exercise may alleviate pain by improving the apoptosis caused by dyslipidemia.

### The role of exercise in the deficit of nitric oxide synthesis

Nerve dysfunction is a common complication of experimental diabetes caused by reduced endothelial function and defective neural blood flow (Mrugacz et al., 2021). This may present clinically as DNP (Callaghan et al., 2012; Calcutt, 2020). Reduced diabetic neural blood flow is caused by hyperglycemia leading to reduce nitric oxide (NO) in vascular endothelial cells and further affecting impaired nitric oxide synthase (NOS). Insulin can increase NO by activating intracellular enzymes phosphatidylinositol-3 kinase (PI3K) and protein kinase B (Akt) to further activate endothelial NOS (eNOS) to increase NO, and restore neurological reactivity associated with neural blood flow (Callaghan et al., 2012). This insulin effect of improving vascular reactivity is disrupted by insulin resistance (Wallis et al., 2002). However, a related study has found that exercise can reverse this damage (Rattigan et al., 2001). To verify that exercise ameliorates decreased vascular nerve reactivity and reduced NO signaling that contributes to peripheral nerve dysfunction, Olver et al. (2014) established a diabetic rat model by STZ injection, performed insulin treatment and exercise training, and then observed motor nerve conduction velocity (MNCV) and eNOS expression in the rat’s sciatic nerve. The results revealed that the diabetes sedentary (DS) rats had lower MNCV than the control sedentary (CS) and diabetes exercise-trained (DX) rats, and that the eNOS expression of the DX rats was remarkably higher than that of the DS rats. These results suggested that motor nerve and vascular nerve functions are impaired in diabetic rats, and that exercise can improve this impairment. The effect of the exercise was found to be higher in the CX rats than in the DX rats in terms of MNCV and eNOS expression. In addition, the expression of MNCV and eNOS was higher in the CX rats than in the DX rats. This suggests that medium to high blood glucose reduces vasa nervorum and nerve function in rats (Olver et al., 2014). Therefore, exercise maybe can reverse the deficit of NO synthesis by reversing the reduction of eNOS expression in nerves caused by diabetes, thus partially restoring peripheral nerve dysfunction and reducing pain.

### The role of exercise in the dysregulation of voltage-gated calcium channel

Pain related to DNP can be relieved by following strict glycemic control (refer to section “The role of exercise in glucose control and IENF” for details). However, in some cases, strict glycemic control alone does not relieve pain (Calcutt, 2002). Dysregulation of ion channel function is thought to be a cellular mechanism of DNP (Gooch and Podwall, 2004). Among them, VGCCs help in mediating neuropathic pain (Julius and Basbaum, 2001; Zamponi et al., 2009). T-type VGCCs involved in the modulation of neuronal excitability (Todorovic and Jevtovic-Todorovic, 2006) have an important role in DNP (Jagodic et al., 2007). Unlike T-type VGCCs, N-type VGCCs are primarily involved in the release of injurious neurotransmitters like glutamate and substance P, thus propagating the pain. To investigate the mechanisms associated with treatment of DNP by exercise through high- and low voltage-activated Ca2 + channel function in DRG neurons, a DNP rat model was established by Shankarappa et al. (2011) by STZ injection and administration of forced exercise. The results showed that the forced exercise remarkably delayed the tactile hypersensitivity (a semiquantitative behavioral measure of painful neuropathy) in DNP rats, and that this may be due to an analgesic mechanism independent of glycemic control because altering blood glucose levels does not change tactile sensitivity in exercising rats. In addition, this study found that exercise significantly increased the dose of naloxone required to achieve tactile hypersensitivity and attenuated diabetes-related changes in the function of VGCCs. Previous studies have shown that the analgesic mechanism of opioid receptors is related to inhibition of VGCC function (Pettinger et al., 2013). These results suggested that exercise may alter the function of VGCC for analgesic purposes in part by altering opioidergic tone.

## Conclusion and outlook

At present, the number of patients with diabetes mellitus is very large, and one-thirds of them present with DNP (Abbott et al., 2011). The pathological mechanism of DNP is still unclear, the above mechanisms have not been fully confirmed, and a large number of RCTs are needed to verify them in the future. Exercise therapy for DNP is receiving increasing attention. Various exercises are effective in improving DNP, including resistance exercise, aerobic exercise, swimming exercise, etc. Naturally, not all exercises have a good analgesic effect. For example, in terms of exercise duration, a short-term exercise may not improve DNP (Cheung et al., 2009); in terms of exercise intensity, a low-intensity exercise has a good analgesic effect (Johnson and Takemoto, 2019). However, excessive exercise intensity reduces IENF, thus leading to severe pain (Do et al., 2020). This review describes the possible mechanisms of exercise in treating DNP through a possible relationship of diabetic neuropathy with the pathological mechanisms of DNP, which provide a basis for future exercise treatment of DNP.

## Author contributions

Y-LZ and BG: draft conception, project administration, and funding acquisition. JL, H-QZ, BG, and Y-LZ: writing, reviewing, and editing. All authors contributed to the article and approved the submitted version.
